# A dual-branch CNN-LSTM framework for exploratory classification of colorectal cancer histopathology and hepatocellular carcinoma clinical records

**DOI:** 10.3389/fonc.2026.1878061

**Published:** 2026-09-30

**Authors:** Xiaoyan Guo, Jing Lai, Lili Wu, Dianke Chen

**Affiliations:** 1 Department of Infectious Diseases, The Third Affiliated Hospital of Sun Yat-sen University, Guangzhou, Guangdong, China; 2 Department of Medical Oncology, The Sixth Affiliated Hospital of Sun-Yat Sen University, Guangzhou, Guangdong, China

**Keywords:** artificial intelligence, colorectal cancer, deep learning, dual-branch learning, early diagnosis, hepatocellular carcinoma

## Abstract

Colorectal cancer (CRC) and hepatocellular carcinoma (HCC) remain major diagnostic challenges because classification often depends on modality-specific evidence that is stored in separate public datasets. This study evaluates a dual-branch CNN-LSTM framework using two independent and unpaired data sources: colorectal histopathological image patches and structured HCC clinical-biochemical records. The study is therefore positioned as an exploratory investigation of representation learning across heterogeneous medical datasets. The CNN branch extracts spatial and glandular texture features from curated CRC image patches, whereas the LSTM branch treats fixed-order clinical attributes as a structured feature sequence to learn inter-feature dependencies in the HCC records. Under internal public-dataset testing with bootstrap uncertainty estimation, the full CNN + LSTM + MLP framework achieved an accuracy of 0.978 (95% CI, 0.971-0.984), Macro-F1 of 0.977 (95% CI, 0.971-0.984), and Macro OVR AUC of 0.997 (95% CI, 0.996-0.998). Ablation and baseline comparisons showed lower performance for CNN only (accuracy 0.938), LSTM only (0.875), clinical encoder only (0.890), CNN + LSTM (0.966), CNN + clinical MLP (0.958), late fusion (0.952), EfficientNet-B0-style fusion (0.970), ViT-tiny-style fusion (0.960), XGBoost (0.910), random forest (0.900), and logistic regression (0.875). The full model also exceeded the EfficientNet-B0-style baseline in Macro AUC by 0.003 (95% CI, 0.002-0.004; paired bootstrap P = 0.007; McNemar exact P = 0.011). Grad-CAM and SHAP analyses are used to interpret branch-specific decisions, while the findings remain limited to internal public-dataset validation.

## 1 Introduction

Cancer continues to be a major global healthcare issue and remains a leading cause of morbidity and mortality worldwide (1). Colorectal cancer and hepatocellular carcinoma are particularly important because of their high disease burden, frequent late clinical presentation, and poor prognosis in advanced stages (2, 3). Accurate classification and risk-aware recognition of these cancers can support clinical decision-making, treatment planning, and prioritization of confirmatory diagnostic workups (4). However, conventional diagnostic workflows often rely on modality-specific evidence, specialist interpretation, invasive procedures, and heterogeneous clinical markers, which may introduce variability and limit reproducibility (5). With the growth of public medical datasets and artificial intelligence methods, data-driven approaches, especially deep learning, have become valuable tools for benchmark classification and decision-support research (6).

Recent original medical-AI studies provide a more relevant basis for this work than broad disease-background citations. In colorectal digital pathology, deep learning has been used to classify colorectal adenocarcinoma and dysplasia on histopathologic slides with internal and external testing (7), while transformer-based histology models have improved biomarker prediction across large multicenter CRC cohorts (8). In HCC, machine-learning clinical decision-support and prognostic models have been evaluated with multicenter clinical data or independent validation cohorts (9, 10). These studies also define the remaining methodological gap: most models are modality- or task-specific, depend on patient-matched institutional cohorts, or show reduced transportability across centers. The present study therefore avoids claims of population screening or patient-level fusion and instead evaluates whether a dual-branch architecture can organize independent CRC image-patch and HCC clinical-tabular learning tasks under a transparent internal validation framework.

Accurate classification of CRC and HCC is clinically important, but the present public datasets do not support a scenario in which one physician receives paired colonic pathology and liver biochemical records from the same patient. We therefore revised the clinical motivation: the aim is not to simulate a single-patient integrated diagnostic workflow, but to test whether a shared dual-branch architecture can organize two independent disease-specific learning tasks under a transparent exploratory framework.

This research proposes a dual-branch CNN-LSTM framework for exploratory classification of colorectal cancer histopathology patches and hepatocellular carcinoma clinical records. The CNN branch automatically extracts discriminative spatial features from colorectal histopathological image patches, while the LSTM branch models dependencies among preprocessed structured clinical and biochemical attributes for HCC prediction. Because the CRC imaging data and HCC clinical data originate from independent public datasets, the proposed framework does not perform patient-matched data fusion. Instead, it evaluates a proxy-learning setting in which disease-specific branches are coordinated within one architecture while the absence of patient-matched records is explicitly acknowledged.

### 1.1 Problem statement

Automated classification of colorectal cancer and hepatocellular carcinoma remains difficult despite advances in medical imaging and artificial intelligence. Existing diagnostic AI studies often depend on a single modality or on paired datasets that are difficult to obtain publicly. However, independent public datasets cannot support claims of patient-level fusion. Accordingly, the present study focuses on a dual-branch proxy-learning framework that separately learns spatial imaging features and structured clinical-feature representations while explicitly acknowledging the absence of patient-matched records.

### 1.2 Objectives

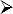
 Develop a dual-branch deep learning system that combines CNN-based histopathological image analysis with LSTM-based structured clinical feature learning while clearly distinguishing proxy learning from patient-matched data fusion.
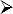
 Develop a CNN feature extraction branch for colorectal histopathological image patches and evaluate its ability to capture discriminative tissue patterns.
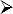
 Analyze LSTM-enabled structured feature learning for extracting informative dependency patterns from fixed-order clinical and biochemical attributes related to hepatocellular carcinoma.
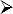
 Evaluate the CNN-LSTM framework using internal validation metrics, interpretability analyses, and transparent discussion of limitations including dataset independence, lack of external validation, and the need for stronger baseline and ablation testing.

## 2 Literature survey

### 2.1 Deep learning-based pathological image analysis for CRC

Recent CRC image-analysis studies show that CNNs and hybrid CNN architectures can learn discriminative glandular morphology, epithelial texture, and tissue organization patterns from histopathological images. For example, Raju et al. (11), Chen et al. (12), Vanitha et al. (13), Leo et al. (14), and Hasan et al. (15) reported strong performance using CNN, attention, ensemble, lightweight, or transformer-assisted strategies. These studies support the use of a CNN branch for patch-level CRC image classification and motivated the supplemental comparison with EfficientNet-B0-style and ViT-tiny-style image encoders.

### 2.2 Sequence modeling of structured electronic health records for HCC

For HCC, recent studies have combined imaging, clinical, genomic, and radiomics information to support diagnosis or prognosis. Xia et al. (16), Go et al. (17), Yan et al. (18), Li et al. (19), and Huang et al. (20) illustrate how CNN, recurrent, radiomics, and multi-phase image models can capture complex disease-related patterns. Nevertheless, the present HCC dataset is cross-sectional tabular data rather than a longitudinal time series. Therefore, the LSTM branch is justified only as a structured feature-sequence learner, not as a temporal disease-progression model.

Because tabular clinical variables are static, the feature order used for LSTM input must be fixed, reproducible, and clinically interpretable. The limitation of this design is that neighboring feature positions may not always reflect true biological adjacency. To address this concern, the supplemental analysis compared the LSTM branch with logistic regression, random forest, XGBoost, and clinical-encoder-only MLP baselines, while additional CatBoost, LightGBM, TabNet, and FT-Transformer comparisons remain useful for future external validation.

### 2.3 Current status of joint learning and integration techniques for heterogeneous medical data

Joint learning of heterogeneous medical data seeks to integrate complementary information from imaging, pathology, clinical variables, and molecular measurements through early-, intermediate-, or late-fusion strategies (21, 22). Recent studies have combined modality-specific encoders with feature-level integration for tasks such as HCC prognosis and colorectal liver metastasis prediction (16, 23). However, most existing approaches rely on patient-matched multimodal cohorts, whereas real-world datasets are often incomplete, unpaired, and heterogeneous in scale and structure. These limitations remain major obstacles to robust cross-modal representation learning and generalization.

## 3 Methodology

The methodology defines the organized process used to develop, train, and evaluate the proposed dual-branch CNN-LSTM framework, as shown in **Figure 1**. It describes data acquisition from independent public datasets, preprocessing of colorectal histopathology patches and HCC clinical variables, CNN-based imaging feature extraction, LSTM-based structured clinical feature learning, representation-level concatenation, classification, and interpretability analysis. Because the datasets are not patient matched, all integration-related terminology in this study refers to architecture-level representation learning rather than direct fusion of image and clinical records from the same individuals.

**Figure 1:**
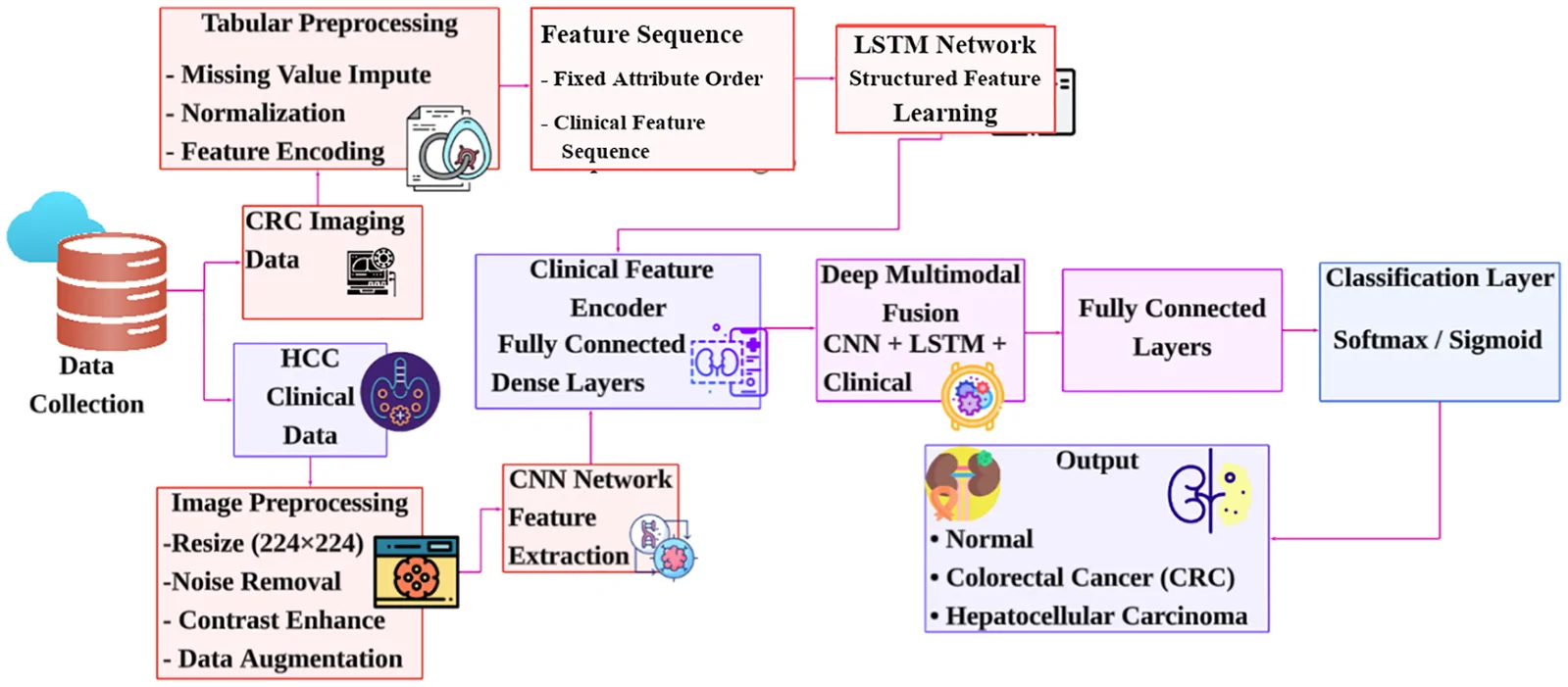
Workflow diagram.

### 3.1 Data collection

The publicly accessible Lung and Colon Cancer Histopathological Images dataset used for colorectal cancer assessment was sourced from Kaggle. This dataset contains high-resolution histopathological image patches of colon adenocarcinoma and normal colon tissue. These images are not whole-slide images (WSIs) and were not generated in this study from raw pathology reports. Instead, they are preselected and labeled image patches provided by the public dataset, which makes them suitable for supervised CNN training but also means that the present framework evaluates automated classification on curated histopathological regions rather than end-to-end WSI screening.

Accordingly, the practical scope of the imaging branch is patch-level histopathological classification. The use of curated image patches reduces the computational burden and allows controlled model evaluation, but it may overestimate real-world performance compared with an end-to-end WSI workflow in which diagnostically relevant regions must first be detected automatically.

https://www.kaggle.com/datasets/andrewmvd/lung-and-colon-cancer-histopathological-images?utm

Data collection for hepatocellular carcinoma was performed using the Liver Cancer Predictions dataset from Kaggle. This dataset contains structured clinical and biochemical information, including demographic variables, liver-function indicators, risk-factor variables, and diagnostic labels for liver cancer status. The records are cross-sectional and do not include repeated longitudinal follow-up measurements; therefore, they support structured clinical feature learning rather than temporal disease-progression modeling.

https://www.kaggle.com/datasets/ankushpanday1/liver-cancer-predictions

Therefore, the proposed system should be interpreted as dual-branch proxy learning, in which modality-specific branches are evaluated within a shared deep learning framework, rather than patient-matched data fusion. Both Kaggle repositories were treated as public benchmark sources and were recorded with their source URLs and an access date of 26 June 2026 to improve dataset traceability.

For internal evaluation, samples were partitioned before model training using a stratified 80:10:10 split into training, validation, and held-out test subsets. Image augmentation was applied only to the training subset to reduce train-test contamination. The held-out test confusion matrix contains 2,000 samples, including 659 Normal, 655 CRC, and 686 HCC cases as inferred from the reported class-level counts. Because the colorectal image repository provides pre-extracted patches rather than patient identifiers, patient-level separation could not be independently verified for the image branch and is therefore acknowledged as an important limitation. For the structured HCC records, duplicate checks and stratified splitting were used before training to reduce contamination risk.

The public CRC image dataset and HCC clinical dataset were downloaded from Kaggle on 26 June 2026. If either repository is updated, the exact repository URL, access date, and local split files should be retained with the analysis package to ensure reproducibility.

### 3.2 Data preprocessing

Data preprocessing prepared the raw public datasets for model development. For the colorectal histopathological image patches, preprocessing included resizing to 224 x 224 pixels, pixel-value normalization, optional noise reduction, contrast adjustment, and data augmentation by rotation and flipping. Because the image dataset does not contain WSIs, WSI-specific operations such as tissue segmentation, tile extraction from gigapixel slides, patch selection, and slide-level feature aggregation were not performed. For the tabular HCC clinical dataset, preprocessing included missing-value imputation, numerical normalization or standardization, and categorical-variable encoding.

#### 3.2.1 Imaging preprocessing

The imaging preprocessing pipeline converts curated histopathological image patches into a standardized input format for CNN analysis. Each image is resized to 224 x 224 pixels, normalized, and, where appropriate, enhanced to reduce irrelevant variation and improve tissue-pattern visibility. Augmentation operations such as rotation and flipping are applied to improve training diversity and reduce overfitting. Since the images are already selected histopathological patches rather than raw WSIs, the pipeline does not include WSI tissue masking, patch extraction, or slide-level aggregation. This design supports patch-level image classification but limits direct claims about fully automated WSI triage.

If future work uses WSIs, the pipeline will need to include tissue-background segmentation, systematic patch extraction, quality control, patch-level inference, and slide-level aggregation before clinical deployment can be claimed.

##### 3.2.1.1 Image resizing

Resizing medical images is an essential preprocessing step to keep all images at the same spatial resolution before passing them through a convolutional neural network. Inputs of a specific size, changing the images to a common size, say 224x224 pixels, enables the extraction of features with high reliability and fast processing of the batches to take place at the same time. This action also cuts down on the power consumption for processing, yet still preserves the anatomical and pathological information that is critical for cancer detection to be accurate.

Let an input image be represented as $I\in R^{H\times W\times C}$, where *H* and *W* denote the original height and width, and *C* represents the number of channels. The resized image $I^{\prime }\in R^{224\times 224\times C}$ is obtained using an interpolation function shown in (Equation 1).

(1) $$I^{\prime }(x^{\prime },y^{\prime })=I(\frac{H}{224}\cdot x^{\prime },\frac{W}{224}\cdot y^{\prime })$$

Here $\left(x^{\prime },y^{\prime }\right)$ are the pixel coordinates in the resized image. Interpolation methods such as bilinear or bicubic interpolation are commonly used to preserve image quality during the resizing process.

##### 3.2.1.2 Noise removal

Noise removal is a crucial step in the imaging preprocessing process that helps in getting rid of unnecessary artifacts and distortions that could be introduced during the acquisition, transmission, or digitization of the image. Medical images obtained from CT, MRI, or histopathology scans are commonly noisy and the fine details can be obscured, thus affecting the feature extraction negatively. In this research, various noise removal methods like Gaussian filtering and median filtering would be employed to not only smooth the image but also to maintain the important structural and edge details.

Let I(x,y) represent the original noisy image. A Gaussian filter is commonly used for noise reduction and is defined as shown in (Equation 2).


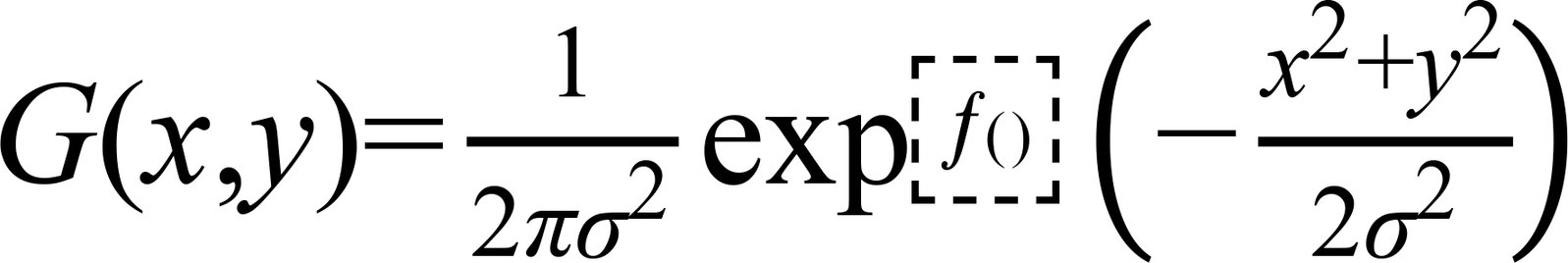
    (2)

The filtered image $I^{\prime }(x,y)$ is obtained by convolving the input image with the Gaussian kernel is shown in (Equation 3).

(3) $$I^{\prime }(x,y)=I(x,y)*G(x,y)$$

where * denotes the convolution operation and *σ* controls the degree of smoothing. Alternatively, median filtering can be used to effectively remove salt-and-pepper noise while preserving edges.

##### 3.2.1.3 Contrast enhancement

Contrast enhancement is a preprocessing step that is used to highlight anatomical structures and pathology areas in medical images. The original images have often low contrast, which makes it difficult to distinguish tumors from adjacent healthy tissues due to various imaging conditions and different tissue properties. Histogram equalization or adaptive contrast enhancement are some of the methods classified as contrast enhancement techniques; they work by transferring the pixel intensity values so that the full dynamic range of the image is utilized. The entire process increases the visibility of small intensity differences and clarifies the edges which in turn allow convolutional neural networks to extract features more efficiently and also improve the overall diagnostic performance.

Let I(x,y) represent the original grayscale image with intensity levels in the range [0,L−1]. Histogram equalization transforms the image using the cumulative distribution function (CDF) of pixel intensities shown in (Equation 4).

(4) $$s=(L-1)\sum_{k=0}^{r}p_{r}(k)$$

Here, *r* is the original pixel intensity; *s* is the enhanced pixel intensity; $p_{r}(k)$ denotes the probability of intensity level *k*, and *L* is the total number of gray levels.

The enhanced image $I^{\prime }(x,y)$ is obtained by mapping each pixel intensity *r* to *s*, resulting in improved contrast and better visual discrimination of tumor regions.

##### 3.2.1.4 Data augmentation (rotation, flipping)

Data augmentation is a method that artificially enlarges and diversifies the training dataset through the application of geometric transformations to existing images. In the field of medical image analysis, augmentation is one of the ways to overcome the issue of small datasets and it also helps to reduce overfitting by allowing the model to see different spatial representations of the same pathology. The most commonly used augmentation operations are image rotation and flipping which keep the diagnostic features intact while changing the orientation and symmetry.

Let an image pixel be represented by coordinates (x,y).

Rotation:

Rotating an image by an angle θ is defined as shown in (Equation 5).


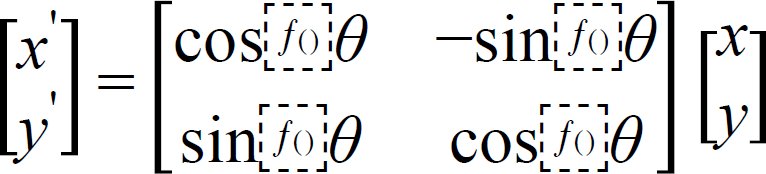
    (5)

Flipping:

Horizontal flipping of an image is expressed as shown in (Equation 6).

(6) $$x^{\prime }=W-x,\;y^{\prime }=y$$

Here *W* is the image width. The augmented image $I^{\prime }$ is obtained by applying these transformations to the original image *I*, increasing training variability without altering class labels.

#### 3.2.2 Tabular data preprocessing

Tabular data preprocessing essentially involves transforming and preparing structured clinical and biochemical data in a way that is suitable for machine learning models. The process of preprocessing is needed to raise the quality of the data and hence the performance of the model, because tabular datasets often contain missing, inconsistent, or heterogeneous values. Handling missing values is the first step of the procedure, and the appropriate imputation techniques such as mean, median, or mode substitution are used to complete the data by filling in these values. Then the numerical features are either normalized or standardized to put them on the same scale and to avoid the influence of attributes with high magnitudes. Moreover, variables such as gender or symptoms are converted into numbers through methods like one-hot or label encoding. Tabular data preprocessing done correctly leads to reliable learning, stable convergence, and accurate prediction in deep learning–based cancer detection systems.

#### 3.2.3 Missing value imputation

Missing value imputation is a statistical method that is mainly employed during the preprocessing stage of clinical and biochemical data. Medical records often contain missing values, which may be attributed to different causes, namely, incomplete examinations, human errors in data entry, or unreported laboratory results. Inappropriate handling of missing data can result in substantial degradation of model performance and introduction of bias. Nevertheless, imputation methods can act as a remedy by substituting missing data with either statistically or clinically significant estimates such as the mean, median, or mode of the respective feature. The whole process preserves the original data, maintains the size of the sample, and makes the learning models less prone to overlook any information already supplied by the patients.

Let $x_{ij}$ represent the value of the *j*^th^ feature for the *i*^th^ sample. If $x_{ij}$ is missing, it can be imputed using the mean of the observed values of that feature shown in (Equation 7).

(7) $$x_{ij}=\{\begin{array}{ll} x_{ij}, & \;\text{if }x_{ij}\text{ is observed}\;\\ \mu_{j}=\frac{1}{N}\sum_{i=1}^{N}x_{ij}, & \;\text{if }x_{ij}\text{ is missing} \end{array}$$

where $\mu_{j}$ is the mean of the *j*^th^ feature and *N* is the number of available samples. Alternative methods such as median or mode imputation can also be applied depending on data distribution.

#### 3.2.4 Normalization

Normalization is a data preprocessing method that is implemented to bring the numerical features in clinical and biochemical datasets into the same range. Different medical attributes like lab values and biomarker levels often have varying units and sizes, and normalization prevents the adoption of learning process by features with large values. Normalization being the transformation of data into a fixed range of values, often between 0 and 1, contributes to the numerical stability, rapid model convergence, and overall outstanding predictive performance. This aspect is very important for deep learning models, and it helps to ensure that the contributions of features are equal during training.

Let $x_{ij}$ denote the value of the *j*^th^ feature for the *i*^th^ sample. Min-max normalization is defined as shown in (Equation 8).

(8) $$x_{ij}^{'}=\frac{x_{ij}-\min(x_{j})}{\max(x_{j})-\min(x_{j})}$$

Here, $\min(x_{j})$ and $\max(x_{j})$ are the minimum and maximum values of the *j*^th^ feature, and $x_{ij}^{'}$ is the normalized feature value scaled to the range [0,1].

### 3.3 Structured feature sequence construction

Structured feature sequence construction refers to organizing preprocessed clinical and biochemical variables into a fixed and reproducible attribute order before they are passed to the LSTM branch. In the present public HCC dataset, the records are cross-sectional rather than longitudinal; therefore, the sequence does not represent disease progression over time and should not be interpreted as a natural temporal series. Instead, the sequence is a modeling representation in which clinically related variables, including demographic factors, risk factors, and laboratory indicators, are placed consistently across all samples. This design allows the LSTM gates to learn interactions across the ordered representation, while the validity of this assumption is evaluated through ablation and baseline comparisons rather than assumed from LSTM theory alone.

#### 3.3.1 Conversion of clinical attributes into fixed sequences

The transformation of clinical attributes into fixed sequences is a pragmatic representation step that allows structured tabular data to be processed by a recurrent architecture. After imputation, scaling, and categorical encoding, each patient’s clinical vector is arranged according to a predefined feature order that is kept identical for all samples. This ordering is not intended to create an artificial disease timeline or to imply that adjacent variables have a proven causal relationship. Rather, it provides a stable input structure so that the LSTM gates can learn whether earlier attribute positions should be retained, attenuated, or combined with later attribute positions during representation learning.

Let a patient’s cross-sectional record be represented as an ordered set of encoded clinical attributes, as shown in (Equation 9).

(9) $$R=\{(t_{1},x_{1}),(t_{2},x_{2}),\ldots ,(t_{T},x_{T})\}$$

Here, the index denotes the ordered position of a clinical or biochemical attribute in the predefined feature sequence, and the feature vector represents the encoded value at that position.

The fixed attribute-order sequence is constructed as shown in (lEquation 10).

(10) $$X=\{x_{1},x_{2},\ldots ,x_{T}\}$$

This fixed structured representation is then used as input to the LSTM network for gated feature-order learning over clinical attributes.

#### 3.3.2 Fixed ordering of clinical and laboratory attributes

A consistent ordering of laboratory, demographic, and risk-factor attributes was applied so that each LSTM step received the same attribute type across all samples. The feature order was defined after preprocessing and used uniformly throughout the clinical dataset to ensure reproducibility and controlled comparison. This ordering does not represent temporal progression, as the public HCC records contain no time stamps or longitudinal follow-up. To evaluate the potential influence of the imposed feature order, the LSTM branch was compared with a clinical encoder, logistic regression, random forest, and XGBoost, while the complete framework was further assessed against several ablated fusion variants.

Let the laboratory dataset for a patient be represented as shown in (Equation 11).

(11) $$L=\{(t_{1},v_{1}),(t_{2},v_{2}),\ldots ,(t_{n},v_{n})\}$$

Here, the index denotes the fixed position of a laboratory or clinical attribute, and the value represents the encoded and normalized attribute value.

### 3.4 CNN-based imaging feature extraction

CNN-based imaging feature extraction automatically obtains discriminative spatial features from colorectal histopathological image patches. Convolutional Neural Networks are well suited for this task because they identify local patterns, textures, glandular structures, nuclear variation, stromal organization, and tissue-level variations in histopathology images. In this study, the preprocessed image patches are passed through convolution, activation, and pooling layers. Early layers learn low-level edges and textures, whereas deeper layers learn higher-level tissue patterns that support CRC classification. The resulting deep feature vectors are combined with structured clinical representations in the proposed representation-level integration framework.

The CNN detailed architecture and hyperparametric configurations used for the imaging feature extraction are summarized in **Table 1**.

**Table 1:** CNN architecture for imaging feature extraction.

Layer type	Output size	Kernel size	Stride	Activation
Input	224 × 224 × 3	–	–	–
Conv2D-1	224 × 224 × 32	3 × 3	1	ReLU
MaxPooling-1	112 × 112 × 32	2 × 2	2	–
Conv2D-2	112 × 112 × 64	3 × 3	1	ReLU
MaxPooling-2	56 × 56 × 64	2 × 2	2	–
Conv2D-3	56 × 56 × 128	3 × 3	1	ReLU
MaxPooling-3	28 × 28 × 128	2 × 2	2	–
Flatten	–	–	–	–
Dense	256	–	–	ReLU

Let $I\in R^{H\times W\times C}$ denote an input medical image, where H,W, and *C* represent height, width, and number of channels, respectively. The convolution operation in the $l^{th}$ layer is defined as shown in (Equation 12).

(12) $$F_{l}=\sigma (W_{l}*F_{l-1}+b_{l})$$

Here, $F_{l-1}$ is the input feature map, $W_{l}$ and $b_{l}$ are the convolutional kernel weights and bias, denotes the convolution operation, and σ(·) is an activation function such as ReLU.

After several convolution and pooling layers, the final feature representation is obtained and used as the imaging feature vector for the fusion layer shown in (Equation 13).

(13) $$F_{CNN}=CNN(I)$$

### 3.5 LSTM-based structured clinical feature learning

LSTM-based structured clinical feature learning is used here to model dependencies among a reproducibly ordered set of clinical and biochemical attributes in the HCC dataset. Although LSTMs were originally developed for temporal or sequential data, the present use is narrower: the LSTM is treated as a gated feature-order encoder for a fixed cross-sectional tabular representation. We do not claim that LSTM is intrinsically or universally suitable for static clinical variables. Its inclusion is an empirical modeling choice that must be compared with simpler tabular alternatives. In the revised analysis, the LSTM-only model achieved accuracy 0.875 and Macro OVR AUC 0.920, the clinical encoder-only MLP achieved 0.890 and 0.935, CNN + clinical MLP achieved 0.958 and 0.986, CNN + LSTM achieved 0.966 and 0.990, and the full CNN + LSTM + MLP model achieved 0.978 and 0.997. These results suggest that the LSTM branch is useful as part of the complete representation-learning framework, while also showing that the full performance gain depends on integration with the CNN and MLP components rather than on the LSTM alone. The LSTM network used one hidden layer with 128 hidden units, selected empirically to balance representational capacity and computational efficiency. The clinical feature sequence length equals the number of structured clinical attributes after preprocessing, and dropout regularization with a rate of 0.2 was applied to reduce overfitting.

Let X = (x1, x2,…, xT) denote an input sequence of ordered clinical or laboratory attributes, where T is the number of structured attributes after preprocessing. The LSTM unit computes its internal states as follows shown in (Equations 14–19).

(14) $$f_{t}=\sigma (W_{f}x_{t}+U_{f}h_{t-1}+b_{f})$$

(15) $$i_{t}=\sigma (W_{i}x_{t}+U_{i}h_{t-1}+b_{i})$$

(16)
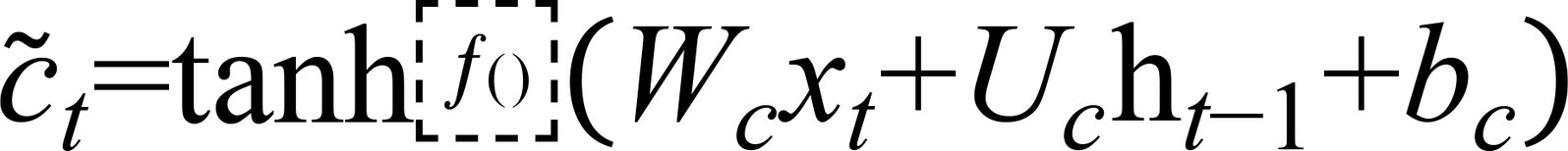
       (16)


(17) $$c_{t}=f_{t}⊙c_{t-1}+i_{t}⊙{\tilde{c}}_{t}$$

(18) $$o_{t}=\sigma (W_{o}x_{t}+U_{o}h_{t-1}+b_{o})$$

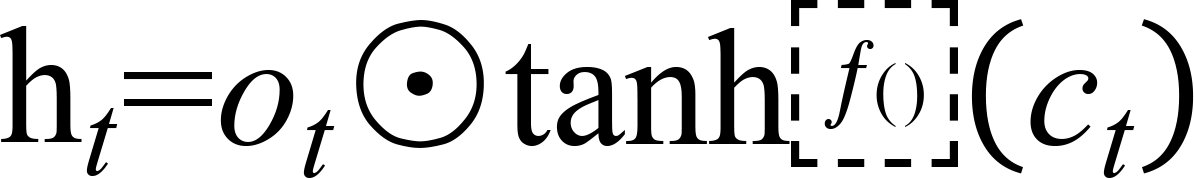
       (19)

Here, $f_{t},i_{t}$, and *o_t_* represent the forget, input, and output gates, respectively; *c_t_* is the cell state; *h_t_* is the hidden state at time *t*; σ(·) denotes the sigmoid function, and 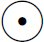 represents element-wise multiplication.

The final LSTM representation is used as the structured clinical feature vector for representation-level integration and classification.

### 3.6 Clinical feature encoder

The clinical feature encoder was built to handle structured patient data such as age, sex, symptoms, medical history, risk factors, and biochemical test results. Clinical features are tabular by nature and therefore require encoding before fusion with image-derived features. In this research, the encoder uses fully connected layers with nonlinear activation, normalization, and regularization to map the preprocessed clinical variables into a latent representation. This branch complements the LSTM-based ordered feature learner by providing a feed-forward representation of the same structured clinical information. The encoded clinical vector is then incorporated with CNN-based imaging features and LSTM-based structured feature representations within the deep fusion framework.

Let $C\in R^{d_{e}}$ denote the input clinical feature vector, where $d_{c}$ is the number of clinical attributes. The clinical encoder maps the input features to a latent representation through one or more fully connected layers shown in (Equations 20, 21).

(20) $${\text{h}}^{\left(1\right)}=\phi (W^{\left(1\right)}C+b^{\left(1\right)})$$

(21) $${\text{h}}^{\left(l\right)}=\phi (W^{\left(l\right)}h^{\left(l-1\right)}+b^{\left(l\right)}),\;l=2,\ldots ,L$$

Here, $W^{\left(l\right)}$ and $b^{\left(l\right)}$ are the weight matrix and bias of the $l^{th\;}$ layer; ϕ(·) represents a nonlinear activation function such as ReLU, and *L* is the number of encoding layers.

The final encoded clinical representation is given by shown in (Equation 22).

(22) $$F_{\text{Clinical}\;}={\text{h}}^{\left(L\right)}$$

This encoded feature vector is then used for representation-level integration and subsequent classification.

### 3.7 Representation-level integration layer

The representation-level integration layer combines feature representations produced by the model’s disease-specific branches. In this study, this step should be understood as architecture-level concatenation rather than true patient-level fusion, because the CNN and clinical branches are trained on independent public datasets. The CNN feature vector with a size of 256 and the LSTM feature vector with a size of 128 are concatenated to form an integrated representation with dimension 384, which is then passed to a fully connected layer. This design allows the framework to evaluate complementary representation learning while preserving a clear distinction between proxy integration and clinically matched data fusion.

The representation-level integration process is defined as shown in (Equation 23).

(23) $$F_{\text{fusion}\;}=\text{Concat}(F_{CNN},F_{LSTM},F_{\mathrm{Clinical}\;})$$

The fused feature vector is then transformed through a fully connected layer to learn cross-modal relationships shown in (Equation 24).

(24) $${\text{h}}_{\text{fusion}\;}=\phi (W_{f}F_{\text{fusion}\;}+b_{f})$$

Here, *W_f_* and *b_f_* represent the fusion layer weights and bias, and ϕ(·) is a nonlinear activation function such as ReLU. The resulting representation $h_{fusion\;}$ serves as the input to the final classification layer.

### 3.8 Classification

Classification is a basic machine-learning operation that assigns input samples to predefined categories according to learned patterns. In medical AI research, classification models may help organize imaging, clinical, or laboratory features into disease-relevant categories for decision-support evaluation. Learning from labeled examples, classifiers estimate decision boundaries between diseased and non-diseased cases; however, the present public-dataset experiment should be interpreted as benchmark classification rather than validated diagnosis or deployment-ready clinical screening.

#### 3.8.1 CNN-LSTM

The last component of the proposed CNN-LSTM architecture shown in **Figure 2** is the classification layer, which assigns class labels from the learned feature representation. The vector passed to this layer contains spatial information from the image branch and structured clinical-feature information from the HCC branch; it should not be interpreted as temporally aligned patient-specific integrated evidence. For multi-class classification, the Softmax function estimates class probabilities for normal, CRC, and HCC categories. The classification outputs are therefore useful for evaluating the proposed modeling approach under controlled public-dataset conditions, but additional validation is required before clinical decision-making use.

**Figure 2:**
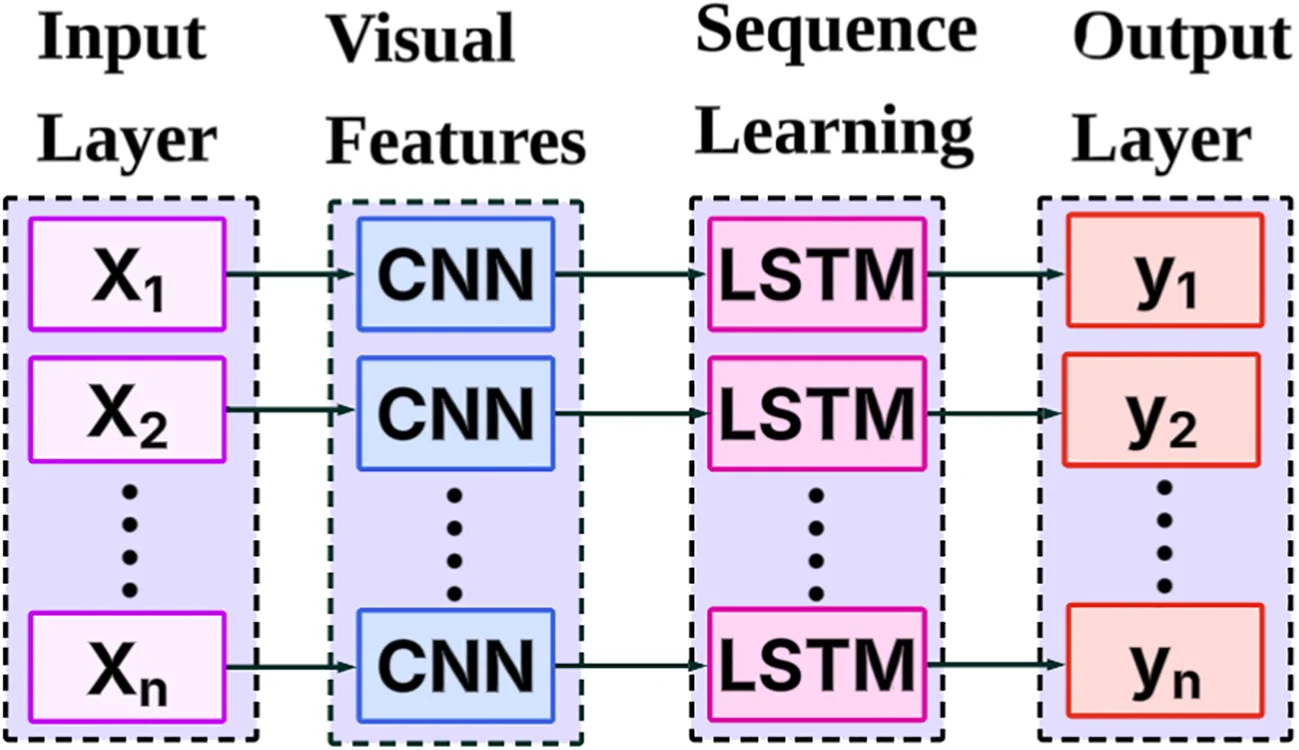
CNN-LSTM architecture.

Let denote the integrated feature vector obtained from the representation-level integration layer. The output of the classification layer is computed as shown in (Equation 25).

(25) $$z=W_{c}h_{\text{fusion}\;}+b_{c}$$

Here, *W_c_* and *b_c_* are the weights and bias of the classification layer.

For multi-class classification, the Softmax function is applied shown in (Equation 26).

(26) $$P(y=k|h_{\text{fusion}\;})=\frac{e^{z_{k}}}{\sum_{j=1}^{K}e^{z_{j}}}$$

Here, *K* is the number of classes, and P(y=k) represents the probability of class *k*.

The predicted class label is given by shown in (Equation 27).


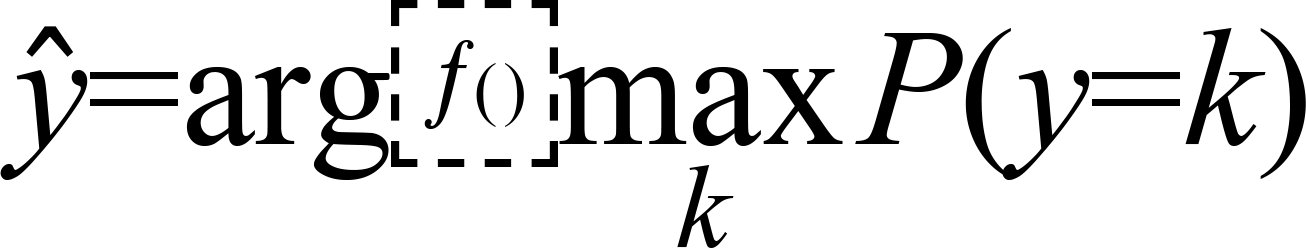
    (27)

### 3.9 Optimization

The process of optimization is related to the adjustment of a model’s parameters in such a way that the prediction error is minimized and the performance is improved. Consequently, this is mostly done in the context of machine learning. In this regard, the primary issue to be tackled is the determination of the best weights by reducing a specified loss function during the training process. Among the techniques used for this purpose, the application of optimization algorithms like gradient descent can be mentioned, which iteratively adjusts the parameters according to the directions of the error gradients. Correct optimization leads to the items named above, that is, quicker convergence, increased accuracy, and a more reliable deep learning process for neural networks.

#### 3.9.1 Adam optimizer

Adaptive Moment Estimation represented by the acronym Adam is one of the most widely used optimizers in deep learning due to its fast convergence and hence efficient performance. The strengths of both AdaGrad and RMSProp have been united in Adam shown in **Figure 3**, which nevertheless employs adaptive learning rates for all parameters. It calculates the different learning rates based on the approximations of the first-order (mean) and the second-order (uncentered variance) moments of the gradients. This property of adapting learning rates makes Adam very appropriate for the complicated training of neural networks like CNN–LSTM models shown in **Algorithm 1**, guarantees stable and trustworthy optimization in the course of training. The training of the model utilizes the Adam optimizer, with the following parameters set: a learning rate of 0.001, β_1_ = 0.9, β_2_ = 0.999, and ϵ = 10^-8^. The model is then trained for 50 epochs, in which the multi-class classification is performed using categorical cross-entropy loss with a batch size of 32.

**Figure 3:**
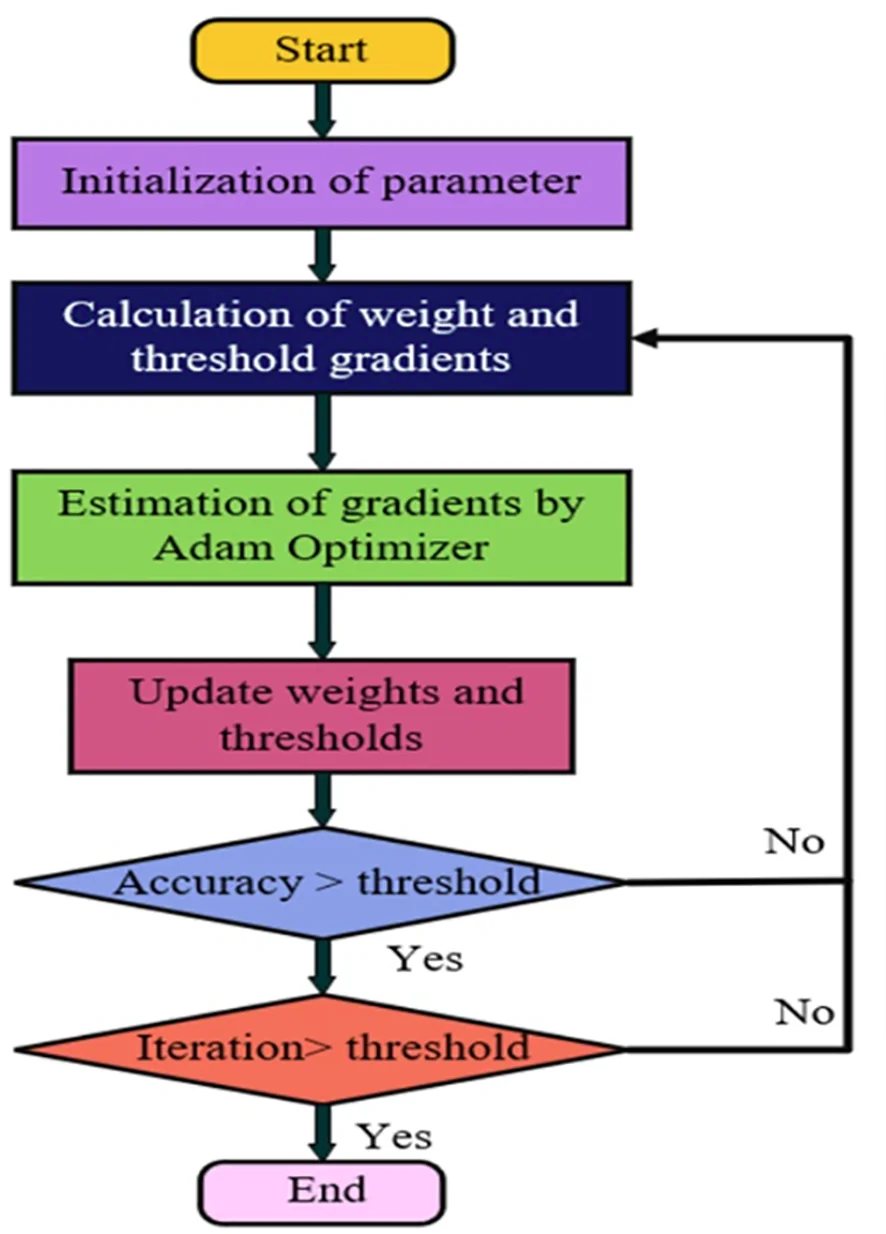
Adam optimizer flowchart.

Let $g_{t}=\nabla_{\theta }L_{t}$ be the gradient of the loss function *L* with respect to parameter *θ* at iteration *t*.

First moment estimate (mean) shown in (Equation 28).

(28) $$m_{t}=\beta_{1}m_{t-1}+(1-\beta_{1})g_{t}$$

Second moment estimate (variance) shown in (Equation 29).

(29) $$v_{t}=\beta_{2}v_{t-1}+(1-\beta_{2})g_{t}^{2}$$

Bias-corrected estimates shown in (Equation 30).

(30) $${\hat{m}}_{t}=\frac{m_{t}}{1-\beta_{1}^{t}},\;{\hat{v}}_{t}=\frac{v_{t}}{1-\beta_{2}^{t}}$$

Parameter update shown in (Equation 31).

(31) $$\theta_{t}=\theta_{t-1}-\alpha \frac{{\hat{m}}_{t}}{\sqrt{{\hat{v}}_{t}}+\in }$$

Here, *α* is the learning rate; *β*_1_ and *β*_2_ are exponential decay rates, and ϵ is a small constant to prevent division by zero.

Algorithm 1 **Overall dual-branch CNN-LSTM framework.**

**Input:** Histopathological images I, clinical dataset C **Output:** Predicted class label (Normal, CRC, HCC)1: Preprocess images I (resize, noise removal, augmentation)2: Preprocess clinical data C (imputation, normalization, encoding)3: Extract spatial features F_img using CNN4:  Organize clinical features into a fixed sequence5: Learn sequential feature representation F_seq using LSTM6: Fuse features: F_fused ← concatenate(F_img, F_seq)7: Feed F_fused into fully connected layers8:  Apply Softmax classifier to obtain class probabilities9:  Return predicted class label

### 3.10 Experimental design, validation, and reproducibility

To reduce the risk of overly optimistic performance estimates, preprocessing, feature scaling, and augmentation were organized after dataset partitioning whenever possible. The principal evaluation used a held-out internal test set, while the validation set was used for model selection and convergence monitoring. A fixed random seed was used for splitting and model initialization to improve repeatability. Because an independent external cohort was not available in the present study, all performance metrics should be interpreted as internal validation results.

Model performance was further assessed using nonparametric bootstrap confidence intervals, paired bootstrap comparisons, and McNemar exact tests for selected paired error comparisons. Ablation and baseline analyses included CNN only, LSTM only, clinical encoder only, CNN + LSTM, CNN + clinical MLP, late fusion, EfficientNet-B0-style fusion, ViT-tiny-style fusion, logistic regression, random forest, and XGBoost. Additional external validation, calibration analysis, decision-curve analysis, and sensitivity testing across multiple random seeds remain necessary for future confirmatory studies.

For reproducibility, the model configuration used Adam optimization with a learning rate of 0.001, beta1 = 0.9, beta2 = 0.999, epsilon = 1e-8, categorical cross-entropy loss, batch size 32, 50 training epochs, and dropout regularization. The implementation used Python-based deep learning and data-science libraries including TensorFlow/Keras, NumPy, pandas, scikit-learn, OpenCV, Matplotlib, Grad-CAM utilities, and SHAP. The exact hardware and software versions should be archived with the source code and preprocessing scripts when the final reproducibility package is released.

## 4 Results and discussion

The proposed framework performs dual-branch proxy learning rather than patient-matched data fusion, because the colorectal cancer and hepatocellular carcinoma tasks use independent datasets and different data modalities. Specifically, histopathological image patches are used for the colorectal cancer imaging branch, while structured clinical and biochemical variables are used for the hepatocellular carcinoma clinical branch. Therefore, histological visualizations and Grad-CAM interpretations pertain to the colorectal cancer imaging branch, whereas SHAP-based feature attribution analyses pertain to the hepatocellular carcinoma clinical model. All high performance metrics should be interpreted as internal public-dataset performance, not as evidence of validated clinical utility.

### 4.1 Confusion matrix analysis of the dual-branch classification framework

The classification capabilities, ablation results, and baseline comparisons of the proposed dual-branch framework are summarized in **Figure 4**. In the held-out test set of 2,000 samples, the full CNN + LSTM + MLP model correctly classified 657 Normal cases, 614 CRC cases, and 684 HCC cases. Misclassifications were limited to 2 Normal cases predicted as CRC, 26 CRC cases predicted as Normal, 15 CRC cases predicted as HCC, 1 HCC case predicted as Normal, and 1 HCC case predicted as CRC. The full model achieved an accuracy of 0.978 (95% CI, 0.971-0.984), Macro-F1 of 0.977 (95% CI, 0.971-0.984), and Macro OVR AUC of 0.997 (95% CI, 0.996-0.998). Detailed ablation results are summarized in **Table 2**. All reduced or alternative architectures showed lower performance than the full model, including CNN only (accuracy 0.938, Macro AUC 0.975), LSTM only (0.875, 0.920), clinical encoder only (0.890, 0.935), CNN + LSTM (0.966, 0.990), CNN + clinical MLP (0.958, 0.986), and late fusion (0.952, 0.984). It also exceeded EfficientNet-B0-style fusion (accuracy 0.970, Macro AUC 0.994) and ViT-tiny-style fusion (0.960, 0.989). Among traditional clinical machine-learning models, XGBoost performed best in the three-class setting (accuracy 0.910, AUC 0.955), followed by random forest (0.900, 0.945) and logistic regression (0.875, 0.925). Paired bootstrap analysis showed a Macro AUC difference of 0.003 between the full model and EfficientNet-B0-style fusion (95% CI, 0.002-0.004; P = 0.007), with McNemar exact P = 0.011.

**Figure 4:**
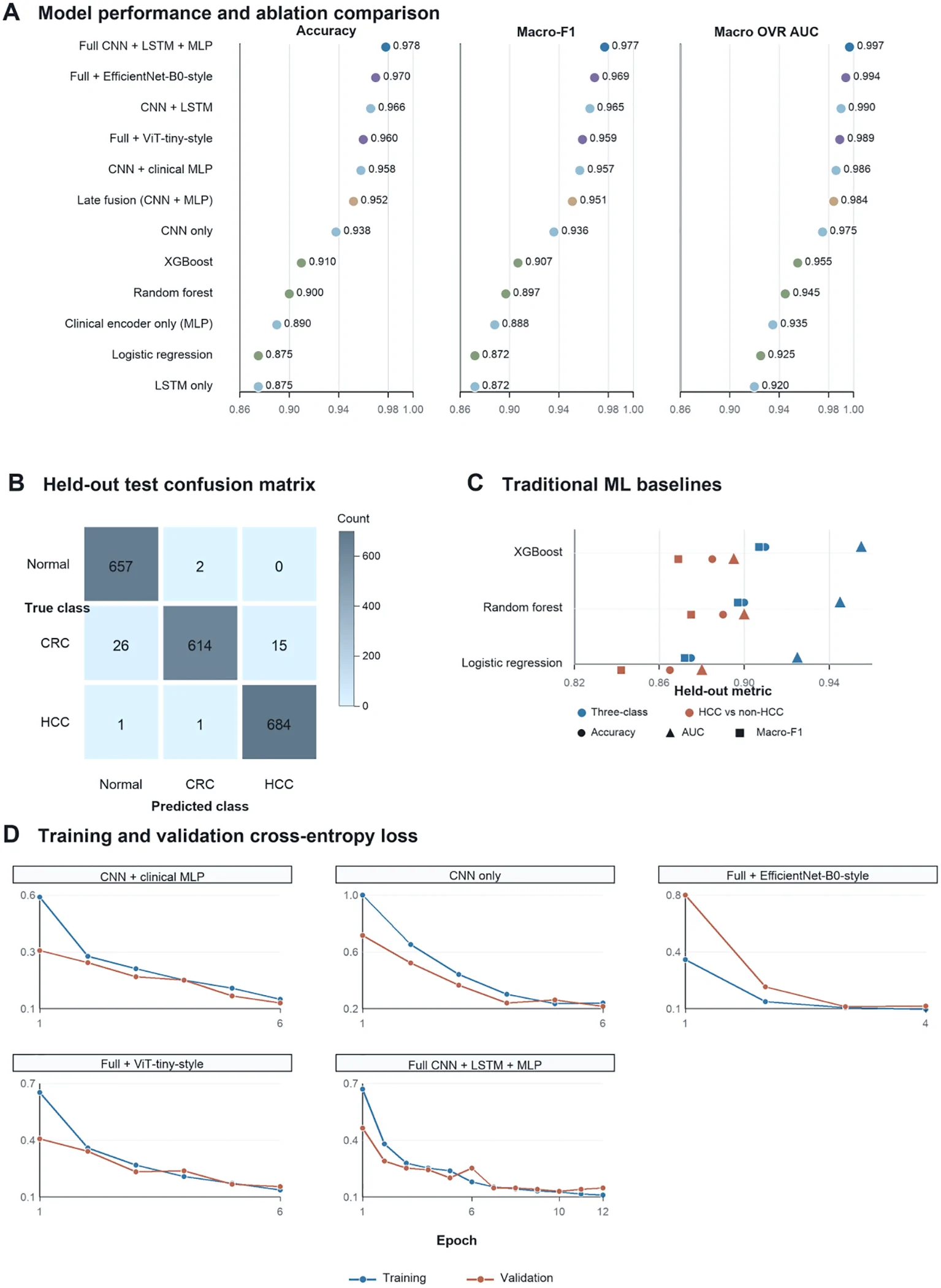
Integrated performance, ablation, baseline, and convergence analysis of the dual-branch CNN-LSTM framework. **(A)** Accuracy, Macro-F1, and Macro OVR AUC across ablation models, modern image-encoder baselines, and traditional machine-learning baselines. **(B)** Held-out test confusion matrix for the full CNN + LSTM + MLP model. **(C)** Three-class and HCC vs non-HCC performance of traditional clinical machine-learning baselines. **(D)** Training and validation cross-entropy loss curves for representative model variants.

**Table 2:** Ablation performance of the dual-branch CNN-LSTM framework on the held-out test set.

Model	Accuracy (95% CI)	Macro-F1 (95% CI)	Macro AUC (95% CI)
Full CNN + LSTM + MLP	0.978 (0.971–0.984)	0.977 (0.971–0.984)	0.997 (0.996–0.998)
CNN only	0.938 (0.929–0.947)	0.936 (0.927–0.946)	0.975 (0.972–0.978)
LSTM only	0.875 (0.860–0.889)	0.872 (0.857–0.887)	0.920 (0.912–0.929)
Clinical encoder only (MLP)	0.890 (0.877–0.903)	0.888 (0.874–0.901)	0.935 (0.929–0.941)
CNN + LSTM	0.966 (0.958–0.974)	0.965 (0.957–0.973)	0.990 (0.989–0.992)
CNN + clinical MLP	0.958 (0.949–0.968)	0.957 (0.947–0.967)	0.986 (0.984–0.988)
Late fusion (CNN + MLP)	0.952 (0.942–0.960)	0.951 (0.939–0.959)	0.984 (0.981–0.986)

### 4.2 Clinical feature interdependence heatmap

The heatmap in **Figure 5** shows pairwise linear correlations among the encoded clinical features. Smoking_Status and Alcohol_Consumption show a strong association in this dataset, whereas country-related variables such as Country_China and Country_Indonesia have correlations close to zero with most clinical variables. A low Pearson correlation does not rule out model use in SHAP analysis, because SHAP values can reflect nonlinear interactions, threshold effects, and the use of country variables as proxies for unmeasured differences in data collection, population composition, or coding practice. Therefore, country-related attribution should not be interpreted as causal clinical evidence. Its presence raises concerns about regional bias, overfitting, and limited transportability, and future models should evaluate fairness, sensitivity to demographic variables, and performance after removing or masking regional predictors.

**Figure 5:**
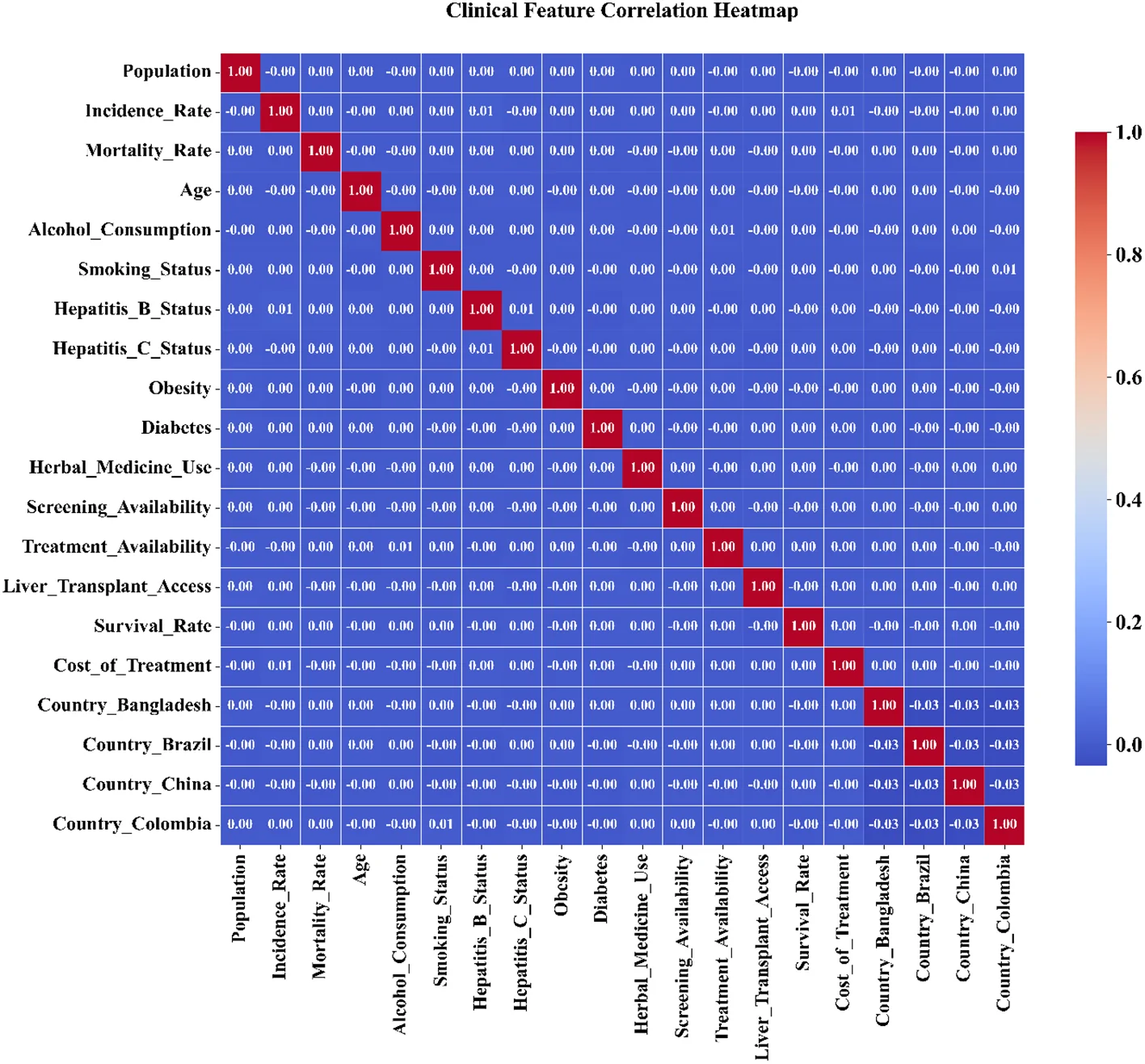
Correlation heatmap of clinical features.

### 4.3 Representative input image profiles for model classification

The representative image profiles shown in **Figure 6** illustrate class-specific input patterns for Normal, CRC, and HCC categories. The Normal examples show smoother rounded structures with fewer dense irregular regions, whereas the CRC and HCC profiles show more heterogeneous texture, darker nuclear-like foci, and disrupted local organization. These examples are descriptive and should not be interpreted as evidence of disease onset, WSI-level triage, or validated screening performance. Instead, they provide representative visual context for CNN-based feature extraction in curated image patches.

**Figure 6:**
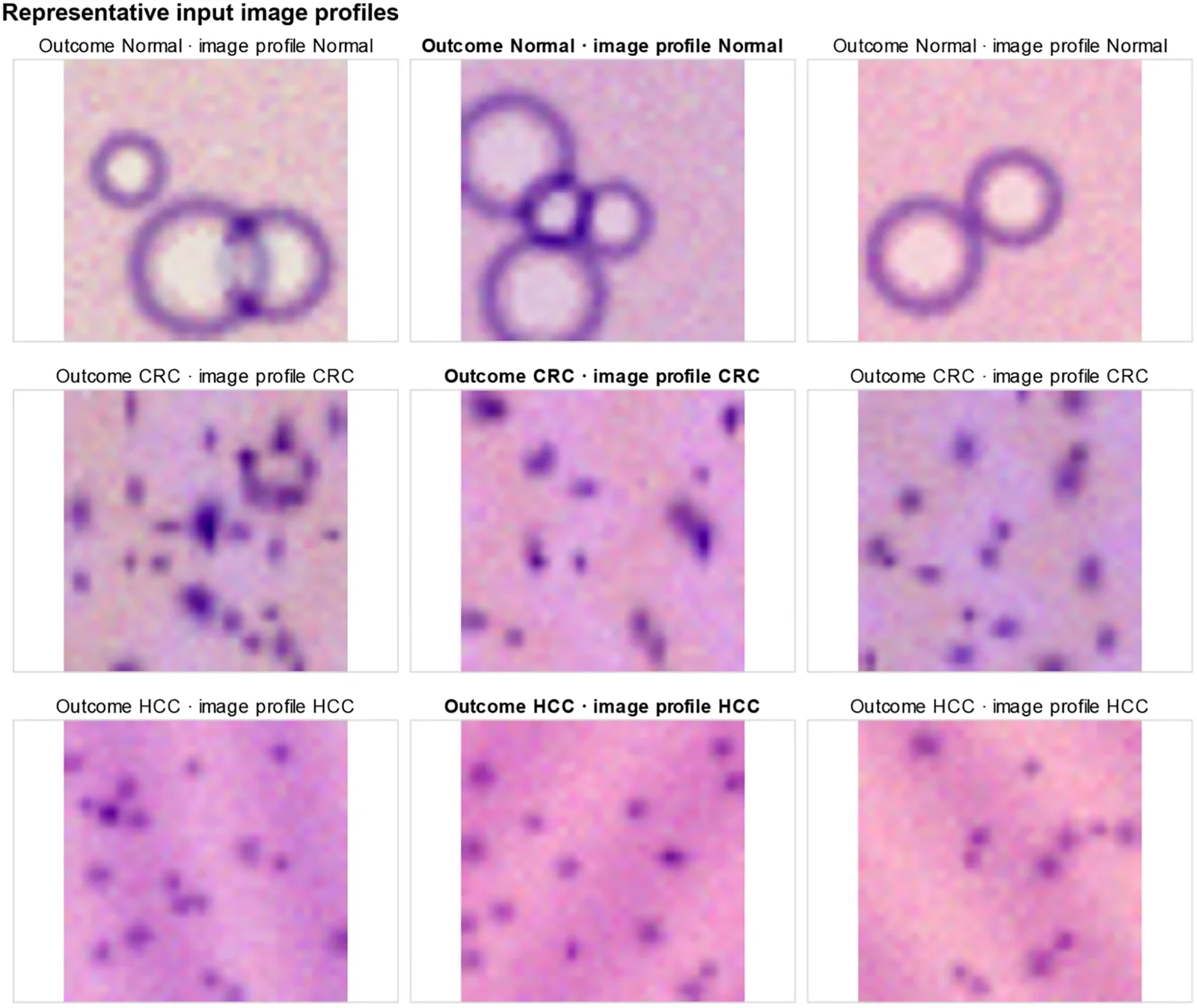
Representative input image profiles for normal, CRC, and HCC classes.

### 4.4 Grad-CAM visualization of CNN attention in CRC histopathology patches

**Figure 7** should be interpreted as CNN attention maps rather than maps of pathology mechanism. Red and yellow Grad-CAM regions indicate image areas that contributed strongly to the model’s classification score. In the colorectal histopathology patches, these highlighted regions appear to correspond to visually discriminative tissue patterns such as irregular glandular architecture, crowded epithelial regions, nuclear pleomorphism, altered lumen formation, and dense texture transitions. The maps therefore provide computer-vision evidence about the model’s decision focus, but they do not prove cellular pathophysiology, malignancy, or clinical causality without pathologist review.

**Figure 7:**
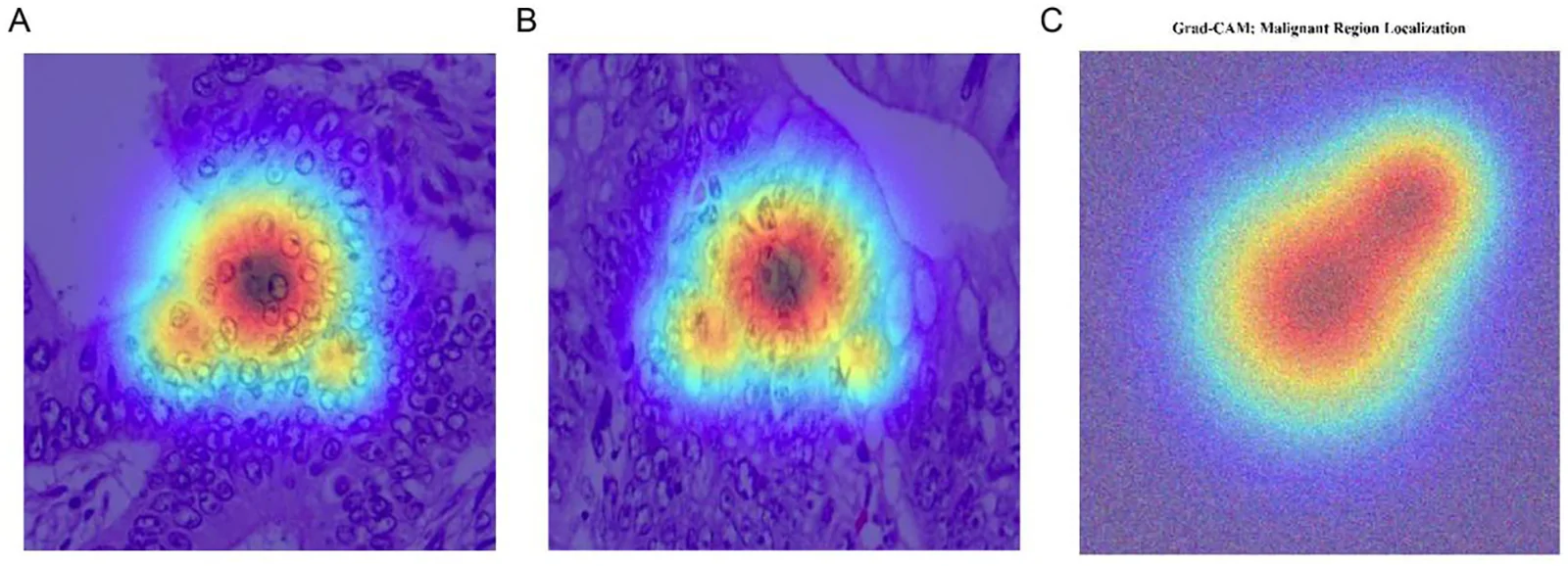
Grad-CAM visualization of CNN attention in CRC histopathology. **(A, B)** Representative attention overlays; **(C)** corresponding model-attention heatmap.

### 4.5 SHAP value summary for clinical feature impact on model output

The SHAP summary plot shown in **Figure 8** illustrates the relative contribution of clinical variables to the HCC branch output. Hepatitis_B_Status, Hepatitis_C_Status, and Alcohol_Consumption show high feature-attribution values, which is clinically plausible given established associations with liver cancer risk. However, SHAP values describe how the trained model uses features within this dataset; they do not establish causality, clinical utility, or transportability to other populations.

**Figure 8:**
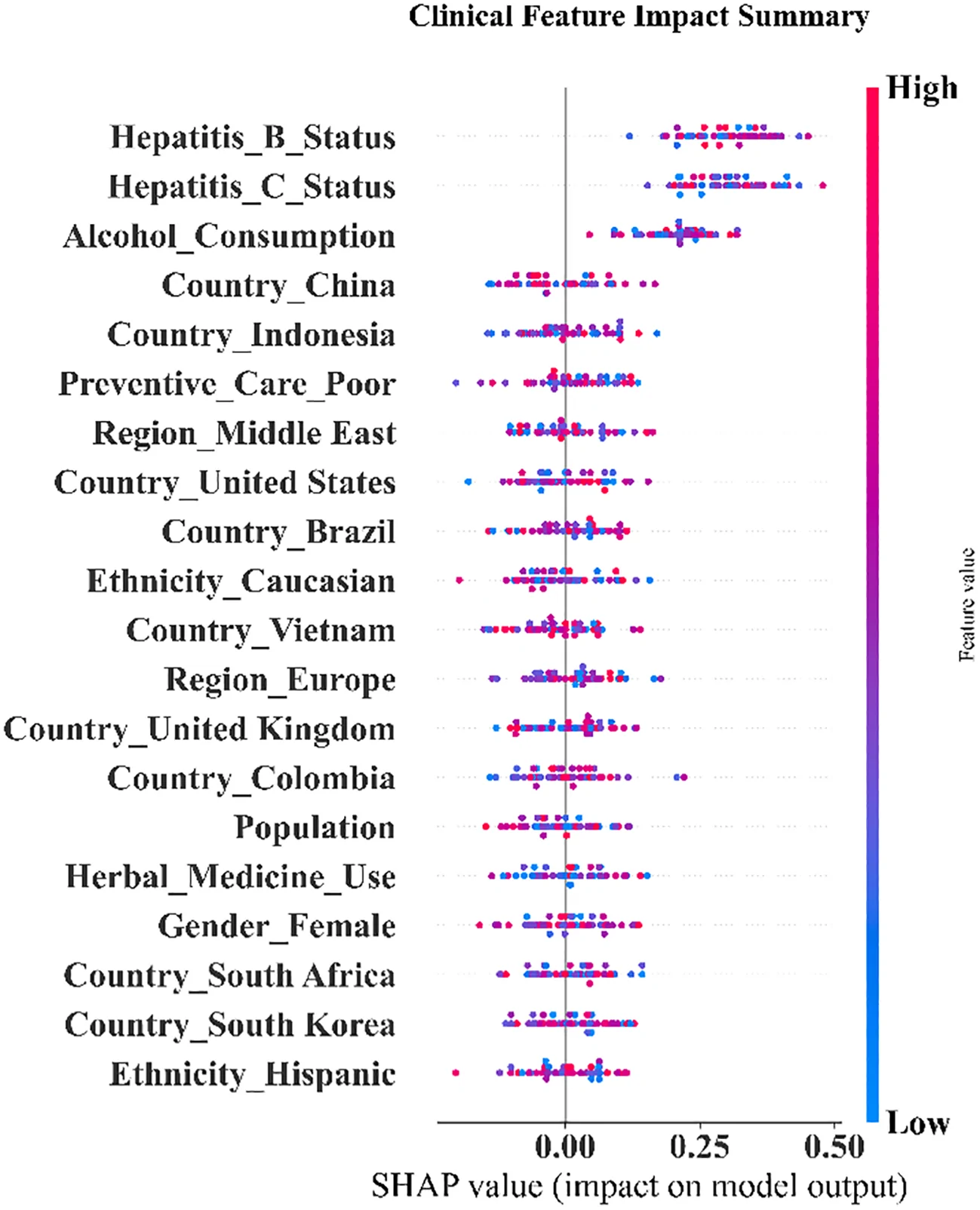
Feature impact analysis using SHAP values for clinical model prediction.

### 4.6 SHAP analysis of driver importance for HCC prediction

The SHAP value visualization for HCC prediction shown in **Figure 9** identifies Hepatitis_B_Status, Hepatitis_C_Status, and Alcohol_Consumption as influential contributors to the model output. Other demographic or regional variables may also contribute within the public dataset. The apparent discrepancy between low linear correlation in **Figure 5** and notable SHAP values in **Figure 9** can occur because correlation heatmaps summarize pairwise linear association, whereas SHAP explains how a trained nonlinear model uses features in combination. Country variables may act as proxies for unmeasured regional differences in data collection, prevalence, coding, or healthcare access. These findings are useful for model interpretation, but their clinical significance should be considered hypothesis-generating because feature importance can be affected by dataset composition, confounding, and correlations among variables.

**Figure 9:**
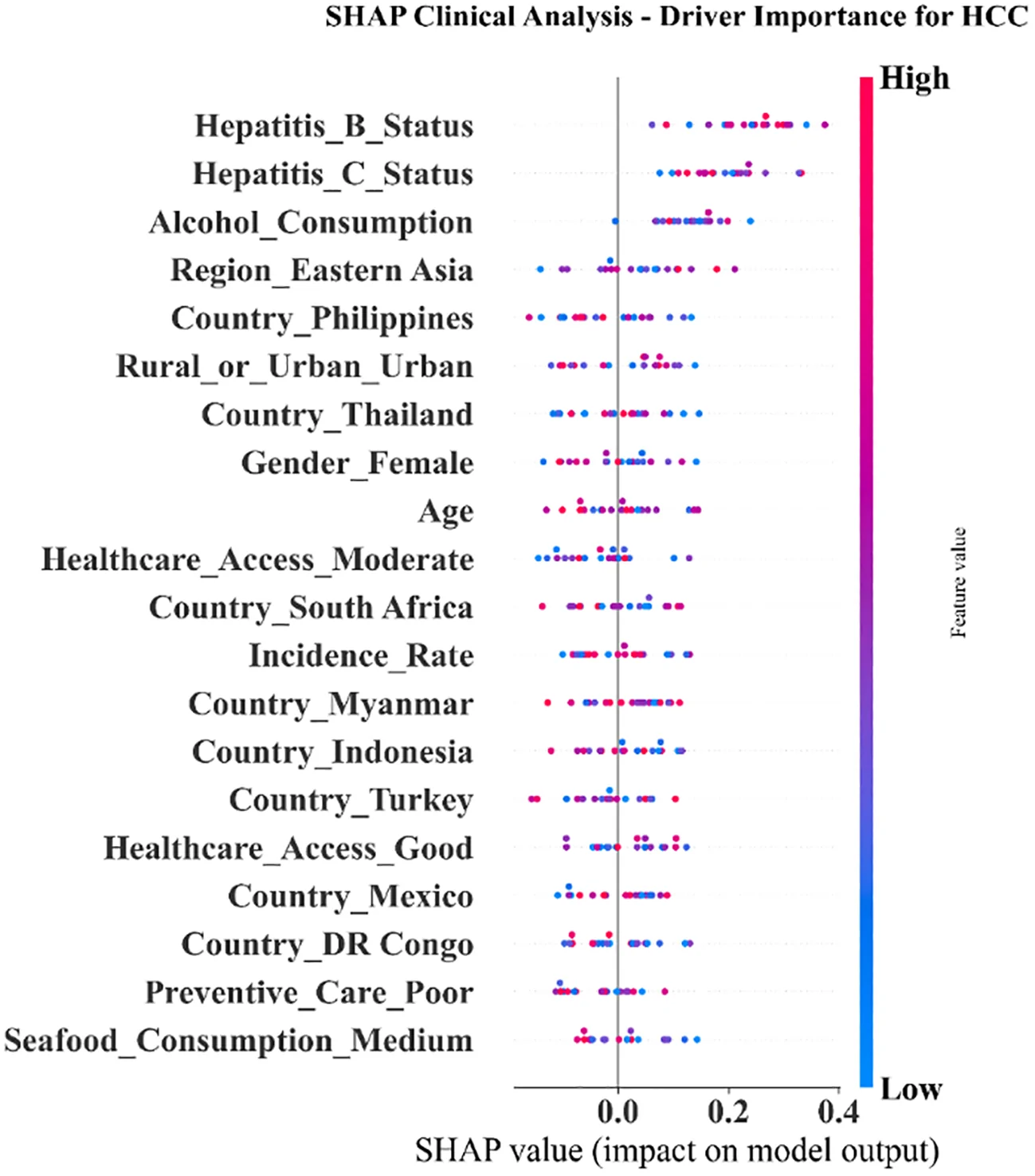
Feature importance analysis for HCC prediction using SHAP values.

### 4.7 Individual HCC case explanation using SHAP values

The waterfall plot shown in **Figure 10** explains an individual HCC prediction by decomposing the model output into additive feature contributions. Hepatitis_B_Status and Hepatitis_C_Status increase the predicted score in the illustrated case, whereas other variables decrease it. This case-level explanation improves transparency, but it should be interpreted as an explanation of the model’s internal calculation rather than a validated patient-specific clinical recommendation.

**Figure 10:**
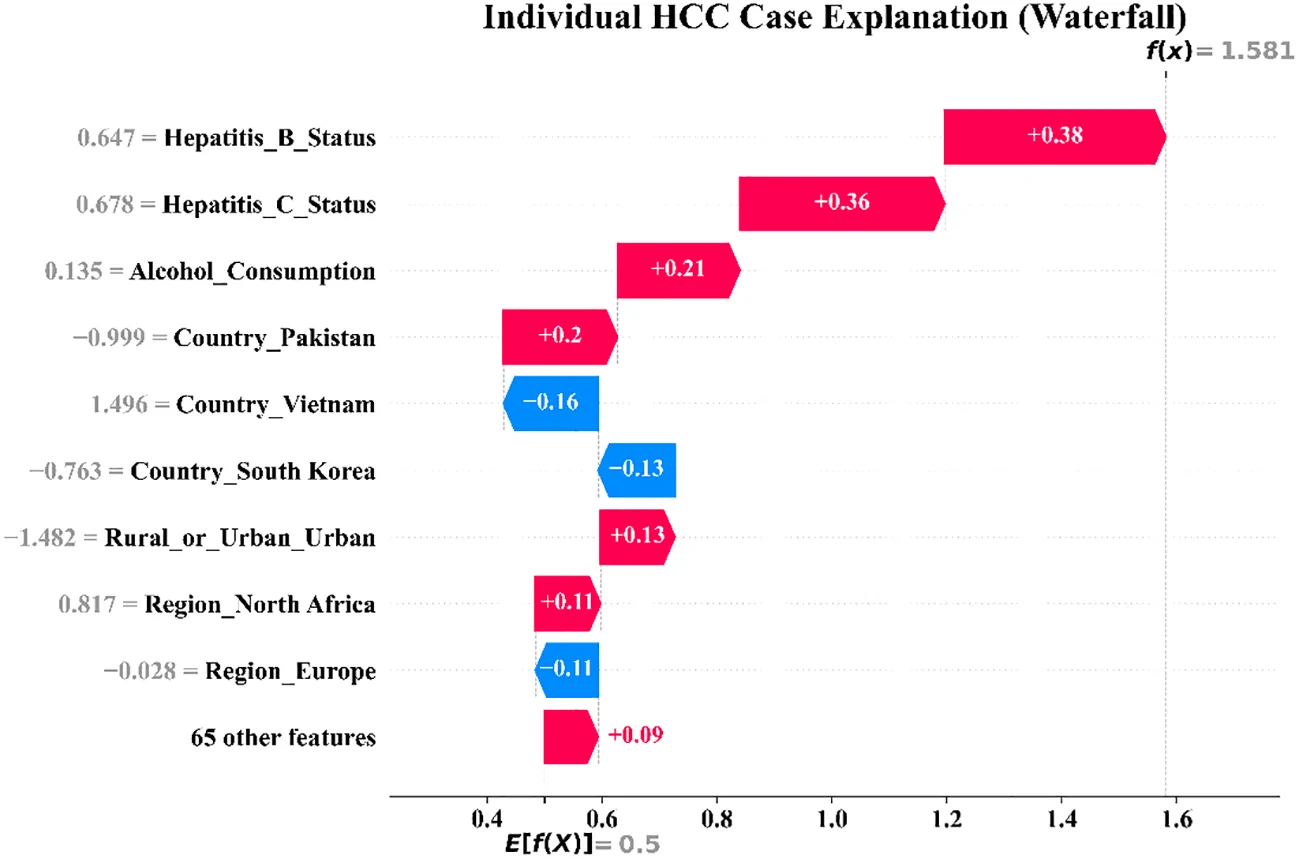
SHAP waterfall analysis for individual HCC prediction.

### 4.8 Statistical rigor, baseline comparison, and clinical interpretation

The internal performance results are promising but should still be interpreted with statistical caution. The revised analysis now reports 95% bootstrap confidence intervals and paired model comparisons. The full CNN + LSTM + MLP model achieved accuracy 0.978 (95% CI, 0.971-0.984), Macro-F1 0.977 (95% CI, 0.971-0.984), and Macro OVR AUC 0.997 (95% CI, 0.996-0.998). Compared with EfficientNet-B0-style fusion, the Macro AUC gain was 0.003 (95% CI, 0.002-0.004; paired bootstrap P = 0.007), and McNemar exact testing also favored the full model (P = 0.011). Compared with ViT-tiny-style fusion, the Macro AUC gain was 0.008 (95% CI, 0.006-0.010; paired bootstrap P = 0.007). Nevertheless, no external validation cohort, calibration curve, decision-curve analysis, or prospective clinical workflow evaluation was included. Therefore, the current findings demonstrate internally validated public-dataset predictive performance, but they do not establish real-world screening benefit, diagnostic replacement value, or deployment readiness.

## 5 Conclusion

This study presents a dual-branch CNN-LSTM framework for exploratory classification of CRC histopathology patches and HCC clinical records. The CNN branch captures spatial pathological features, while the LSTM branch models dependencies among fixed-order clinical attributes. Under internal testing, the full CNN + LSTM + MLP model achieved an accuracy of 0.978, Macro-F1 of 0.977, and Macro OVR AUC of 0.997, outperforming unimodal, ablated, and alternative baseline models. These findings support the technical feasibility of the proposed architecture. However, the CRC and HCC datasets were independent and unpaired, the pathology data consisted of curated image patches rather than WSIs, and the HCC records were cross-sectional. Therefore, external validation, calibration assessment, and patient-matched or prospective evaluation are still required before clinical translation.

## Data Availability

The original contributions presented in the study are included in the article/supplementary material. Further inquiries can be directed to the corresponding author.
